# Study of the Membrane Activity of the Synthetic Peptide ∆M3 Against Extended-Spectrum *β*-lactamase *Escherichia coli* Isolates

**DOI:** 10.1007/s00232-024-00306-3

**Published:** 2024-02-05

**Authors:** Estefanía Fandiño-Devia, Gloria A. Santa-González, Maria C. Klaiss-Luna, Marcela Manrique-Moreno

**Affiliations:** 1 Chemistry Institute, Faculty of Exact and Natural Sciences, University of Antioquia, A.A. 1226, Medellin, 050010 Colombia; 2 Grupo de Investigación e Innovación Biomédica, Facultad de Ciencias Exactas y Aplicadas, Instituto Tecnológico Metropolitano, A.A. 54959, Medellín, 050010 Colombia

**Keywords:** Antimicrobial peptides, Antimicrobial resistance, Extended-spectrum *β*-lactamase-producing in *Escherichia coli*, Fluorescence microscopy, Infrared spectroscopy

## Abstract

*Escherichia coli* is the most common microorganism causing nosocomial or community-acquired bacteremia, and extended-spectrum *β*-lactamase-producing *Escherichia coli* isolates are identified worldwide with increasing frequency. For this reason, it is necessary to evaluate potential new molecules like antimicrobial peptides. They are recognized for their biological potential which makes them promising candidates in the fight against infections. The goal of this research was to evaluate the potential of the synthetic peptide ΔM3 on several extended-spectrum *β*-lactamase producing *E. coli* isolates. The antimicrobial and cytotoxic activity of the peptide was spectrophotometrically determined. Additionally, the capacity of the peptide to interact with the bacterial membrane was monitored by fluorescence microscopy and infrared spectroscopy. The results demonstrated that the synthetic peptide is active against *Escherichia coli* isolates at concentrations similar to Meropenem. On the other hand, no cytotoxic effect was observed in HaCaT keratinocyte cells even at 10 times the minimal inhibitory concentration. Microscopy results showed a permeabilizing effect of the peptide on the bacteria. The infrared results showed that ΔM3 showed affinity for the lipids of the microorganism’s membrane. The results suggest that the ∆M3 interacts with the negatively charged lipids from the *E. coli* by a disturbing effect on membrane. Finally, the secondary structure experiments of the peptide showed a random structure in solution that did not change during the interaction with the membranes.

**Graphic Abstract:**

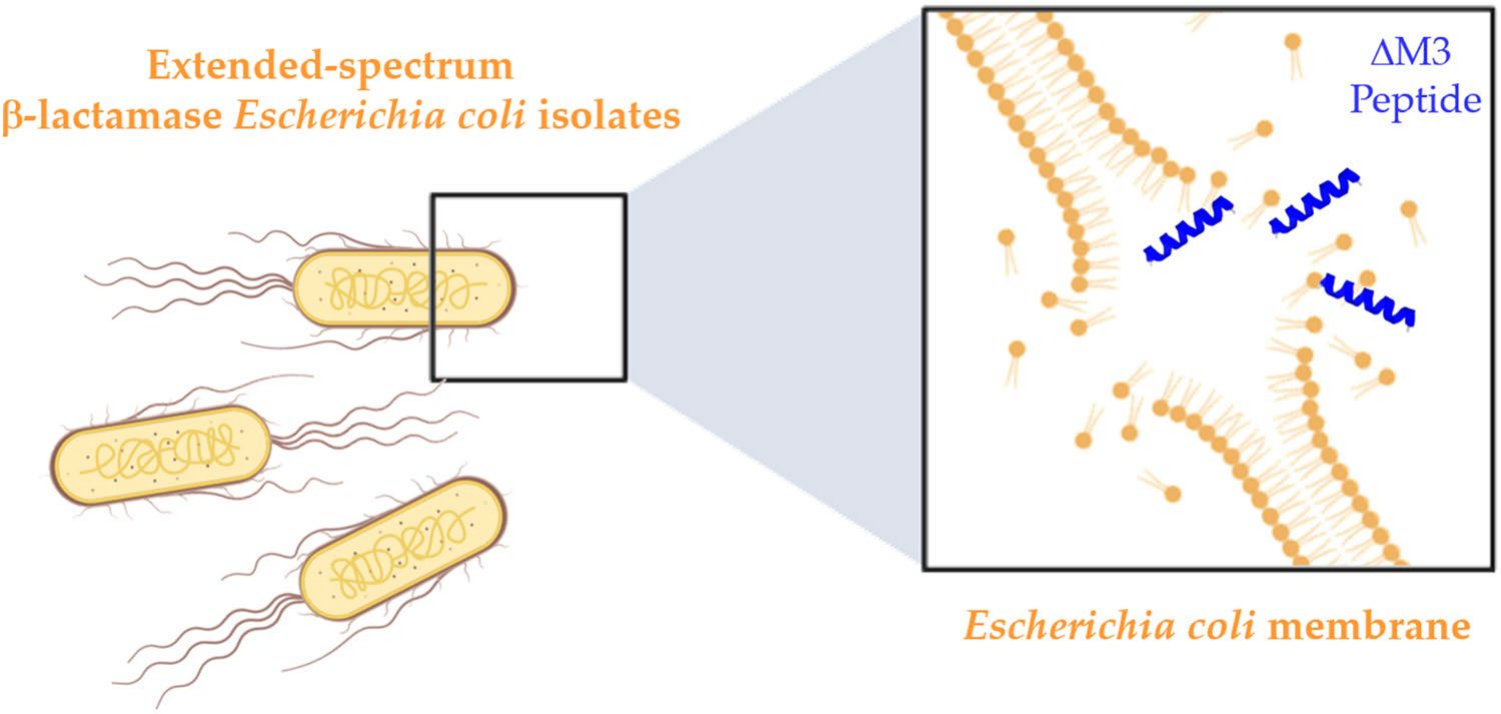

## Introduction

*Escherichia coli* (*E. coli*) is a Gram-negative bacteria widely associated with infections in intensive care units (ICUs), such as ventilator-associated pneumonia, catheter-related blood infections, and urinary tract infections, among others (Sannathimmappa et al. 2021; Sivakumar et al. 2021; Streicher 2021). In this pathogen several defense mechanisms have been characterized, making it efficiently resistant to multiple drugs (Iredell et al. 2016; Poirel et al. 2018; Reygaert 2017). In the last decade, increasing antimicrobial resistance in *E. coli* has led to the spread and dissemination of isolates resistant to almost all currently available antibiotics, leaving few therapeutic alternatives, which has led to a significant increase in mortality rates associated with nosocomial infections, and therefore become a public health problem worldwide (Sannathimmappa et al. 2021; Streicher 2021). Extended-spectrum *β*-lactamases (ESBLs) refer to a group of *β*-lactamases that have acquired the capacity to hydrolyze penicillins, third-generation cephalosporins, and monobactam antibiotics. These enzymes that hydrolyze *β*-lactam antibiotics are derived by mutation from the *β*-lactamases present in most enterobacteria, most frequently in *Klebsiella pneumoniae* and *E. coli* (Bedenić & Meštrović, 2021; Sawatwong et al. 2019). The antibiotic multiresistance profile expressed by these isolates causes, especially in the hospital environment, notable therapeutic problems such as significant delays in the start of effective antibiotic treatment, longer hospitalizations, and higher overall hospital costs than controls with infections produced by non-ESBL strains (Lautenbach et al. 2001). In addition, ESBL production has a significant impact on mortality in pediatric patients (Kim et al. 2002). For these reasons, and given the few treatments available to counteract these infections, ESBLs are considered a critical priority according to the World Health Organization (WHO) (Bezabih et al. 2022).

The search for new antibiotics and their implementation in clinical treatment is a slow and expensive process that in many cases is considered inefficient (Yusuf et al. 2021). According to the WHO, in the near future, the difficulty of treating a bacterial infection will depend on the combination of multi-resistant isolates and the availability or lack of new effective antibiotics (Iredell et al. 2016). Therefore, numerous investigations have focused on evaluating and characterizing new potential molecules known as antimicrobial peptides (AMPs). These molecules have demonstrated broad biological activity against microbes, fungi, parasite viruses, and in the last decade, cancer cells (Lee et al. 2019a; Li et al. 2021; Martínez-Culebras et al. 2021; Nogrado et al. 2022; Tornesello et al. 2020). Additionally, it has been demonstrated that these molecules present a mechanism hard to counter that does not allow the bacteria to easily develop resistance pathways, making the peptides potential therapeutic agents for multi-drug resistant bacteria (Zhang and Falla 2006). AMPs are part of the innate immune system of numerous organisms, exerting their activity through different mechanisms (Tacconelli et al. 2018). The most widely described of these is the irreversible alteration of the bacterial membrane through the electrostatic interaction of the positive charges of the peptide with the anionic groups present in the phospholipids of the membrane, which induces the alteration and ultimately the death of the bacteria (Li et al. 2020; Pane et al. 2017; Travkova et al. 2017; Yan et al. 2021).

Our research focuses on evaluating a synthetic cationic sequence called ΔM3, a cecropin D-derived peptide, against ESBL-producing *E. coli* isolates. We had previously reported the potential of this peptide against *Staphylococcus aureus* (Manrique-Moreno et al. 2021), and its fungicide activity against the yeast forms of *Candida albicans*, *Candida tropicalis*, and *Candida parapsilosis* (Guevara-Lora et al. 2023). Based on the activity of ΔM3, we decided to evaluate its potential antimicrobial and bactericidal activity against several ESBL-producing *E. coli* isolates. Also, with the aim of studying the structure-activity relationship, the 3D predicted structure of ΔM3 was compared with the secondary structure of the peptide in solution and during the interaction with synthetic membranes representative of *E. coli*. Additionally, permeability experiments by fluorescence microscopy and phase transition studies by infrared spectroscopy were performed in order to confirm whether ΔM3 exerts its mechanism of action by altering the membrane structure of the bacteria. Finally, in order to evaluate the potential use of the peptide, cytotoxic experiments with human keratinocyte cells were performed. The results obtained in this research showed that the synthetic peptide ΔM3 is a promising molecule for the treatment of infections caused by ESBL-producing *E. coli*.

## Materials and Methods

### Bacterial Isolates and Growth Conditions

ESBL-producing *E. coli* HD7, HD8, HD11 BK43028, EB101, EB107, and EB127 isolates from the strain bank of the Basic and Applied Microbiology Group (MICROBA) of the University of Antioquia were used. These strains were characterized phenotypically and genotypically and identified as resistant to monobactam antibiotics and to penicillins and cephalosporins up to fourth generation. The *E. coli* ATCC 25922 strain, sensitive to all *β*-lactam antibiotics, was used as a control for the study. The *E. coli* isolates were plated by depletion on agar nutritive (Merck Millipore, Darmstadt, Germany) and were left to incubate at 37 °C for 24 h. After this, 3 to 5 colonies were taken in cation adjusted Müeller Hinton broth (Merck Millipore Darmstadt, Germany), to form a 0.5 McFarland suspension (1 × 10^8^ CFU/ml) for the experiments.

### Lipids and Peptide Synthesis

1-palmitoyl-2-oleoyl-glycero-3-phosphocholine (POPC, Lot. 850457P-500MG-A-211), 1-palmitoyl-2-oleoyl-sn-glycero-3-phosphoethanolamine (POPE, Lot. 850757P-500MG-B-151), sphingomyelin egg chicken (SM, Lot. 860061P-25MG-A-116), 1-palmitoyl-2-oleoyl-*sn*-glycerol-3-phosphoglycerol sodium salt (POPG, Lot. 160-181PG-135) were purchased from Avanti Polar Lipids (Alabaster, AL, USA). HEPES was purchased from Sigma-Aldrich (St. Louis, MO, USA).

According to the ΔM3 sequence (NFFKRIRRAGKRIRKAIISA, Lot. 7215960005/PE6969) 20 mg of the peptide was synthesized without modifications at the N or C-terminus by solid phase synthesis (SPPS) method, having been purchased from GenScript (Piscataway Township, NJ, US). Reverse phase high-performance liquid chromatography (RP-HPLC) was used for the purification of the peptide (> 95%), using a Vydac C-18 preparative column and a mixture of (A) H_2_O with TFA 0.1% (v/v) and (B) acetonitrile (ACN) with TFA 0.1% (v/v) as mobile phase. For elution, the following gradient was used: 30 min with 5–70% B at a flow rate of 1 ml/min and a detection wavelength of 220 nm. The molecular weight of the purified peptide was determined by MALDI-TOF mass spectrometry (MS).

### Antimicrobial Activity Assay

The determination of the minimum inhibitory concentration (MIC) was carried out using the broth microdilution technique, adapting the protocol reported in the guidelines of the Clinical Laboratory Standard Institute (CLSI) (CLSI 2019). For this purpose, 50 µl of the bacterial suspension (0.5 McFarland suspension) of each isolate and 50 µl of the peptide solution in PBS at 40, 20, 10, 5, 2.5, 1.25, 0.625 and 0.312 µM were placed a 96-well polypropylene plate. Subsequently, the antibiotic Meropenem (Thermo Fisher Scientific, Massachusetts, United States) was used as a positive control at a concentration of 2 µg/ml (5.2 µM) and PBS was used as a negative control. The reading of the dishes was carried out for 24 h at 37 °C in a Multiskan-Go spectrophotometer (Thermo Fisher Scientific, Massachusetts, United States). To guarantee the reproducibility of the assay, five independent experiments were performed on each isolate for the different concentrations of the peptides. The determination of the minimum bactericidal concentration (MBC) was made in order to identify the minimum concentration of the peptide to cause the death of 99% of the *E. coli* isolates. For this, 20 µl was taken from the wells where no bacterial growth was evident in the plate microdilution technique, sown by isolation in nutrient agar, and left to incubate at 37 °C for 24 h. Subsequently, the colony-forming units (CFU) were counted.

### Bactericidal Effect of the ∆M3 Peptide on ESBL-Producing *E. coli*

In order to determine the bactericidal activity of ∆M3 on ESBL-producing *E. coli* isolates, strains in the logarithmic growth phase were inoculated in LB culture broth at a volume ratio of 1:100. ∆M3 was added to the bacterial inoculum at final concentrations of 0, 1.25, 5, 7.5, and 10 µM, then incubated at 37 °C with shaking at 200 rpm. Then, 100 µl of bacterial solution from each group was removed after 0, 2, 4, 6, 8, 10, and 12 h of incubation and placed in 96-well microtiter plates. Absorbance values were detected with a Multiskan-Go spectrophotometer at 650 nm (Thermo Fisher Scientific, Massachusetts, United States). To ensure the reproducibility of the assay, three independent experiments were performed.

### Cytotoxic Activity of ∆M3 Against Epidermic Cells

To study the potential cytotoxic effect of ΔM3, HaCaT (human keratinocyte cell line, CLS 300493) cells were cultured in Dulbecco’s modified Eagle medium (DMEM) supplemented with antibiotics (100 U/ml of penicillin, 100 μg/ml of streptomycin) and 10% fetal calf serum at 37 °C in a humidified chamber containing 5% CO_2_. The percentage of cell cytotoxicity was evaluated by MTT according to International Organization for Standardization 10993-5 (Standard 2009). A total of 2.5 × 10^4^ cells/well were seeded onto a 96-well plate, and then incubated for 24 h. Then different concentrations of the peptide were added to the wells, after which the cells were incubated for an additional 24 h at 37 °C. Subsequently, 50 μl of MTT at a concentration of 10 mg/ml was added to each of the wells, after which the cells were incubated for an additional 2 h. The supernatants were then aspirated, and 100 μl of acid isopropanol was added to the wells to dissolve any remaining precipitate. Absorbance was then determined in a multimode Varioskan lux multi-plate reader.

### Evaluation of the Secondary Structure ∆M3 by Infrared Spectroscopy

∆M3 peptide solution was prepared at a 2 mM concentration in buffer (10 mM HEPES at pH 7.4). Two lipid systems were prepared, and appropriate amounts of POPC:SM:POPE (46:42:12; w/w) for the erythrocyte membrane (Olver 2022), and POPE:POPG (70:30; w/w) for *E. coli* lipid system (Nowotarska et al. 2014), were weighted in order to obtain a 6 mM final concentration of the representative liposomes. The lipid systems were dissolved in pure chloroform in a glass test tube, the solvent was evaporated under a stream of nitrogen, and the traces were removed by keeping the samples under reduced pressure (about 13.3 Pa) during 30 min. Dried lipid films were hydrated in buffer. Small unilamellar vesicles (SUVs) were formed by sonicating the samples above the main phase transition temperature of the lipids for 30 min. To determine the secondary structure, ∆M3 was added to the liposome suspension to obtain a peptide-to-lipid molar ratio of 15 mol%. The experiments were performed at 37 °C in an AquaSpec Cell integrated with a Tensor II spectrometer (Bruker Optics, Ettlingen, Germany) with a liquid nitrogen MCT detector using a spectral resolution of 4 cm^−1^ and 120 scans per spectrum. The secondary structure elements α-helices and β-sheets were predicted following the methods supplied by the Confocheck™ system (Bruker Optics, Ettlingen, Germany). These methods were used to calculate the secondary structure using a multivariate partial least squares (PLS) algorithm based on a calibration dataset of 43 proteins.

### Membrane Permeability Assays by Fluorescence Microscopy

In order to determine the effect of the peptides on the ESBL-producing *E. coli* membrane, the fluorescence microscopy technique using SYTOX®Green dye (Thermo Fisher Scientific, Massachusetts, United States) was used. The ESBL-producing *E. coli* H8 isolate was resuspended in trypticase soy broth (Merck Millipore, Massachusetts, United States) and shaken for 18 h at 37 °C. Subsequently, 300 µL of the bacterial inoculum was taken and centrifuged at 2800 g for two minutes. The supernatant was discarded and the pellet was resuspended in 300 µL of sterile deionized water. Subsequently, 5 µl of the SYTOX®Green solution (5 µM) and 20 µl of the solution of ∆M3 peptide were added at the following concentrations: 1.25, 2.5, and 5 µM. The samples were left in the dark and incubated at 37 °C for one hour. Image acquisition was performed through an inverted fluorescence microscope (Axio Observer A1, Carl Zeiss, Germany) with a Mercury lamp (HXP 120 V, Carl Zeiss, Germany). The wavelength was selected using a set of filters to excite the indicator between 450 and 490 nm and obtain emission above 515 nm.

### Evaluation of the Effect of ∆M3 on the Phase Transitions of the Lipid Systems

Supported lipid bilayers (SLBs) of *E. coli* lipid system POPE:POPG (70:30) were prepared in situ in a BioATR II cell integrated to a Tensor II spectrometer (Bruker Optics, Ettlingen, Germany) with a liquid nitrogen MCT detector using a spectral resolution of 4 cm^−1^ and 120 scans per spectrum. The temperature range was set by a Huber Ministat 125 computer-controlled circulating water bath (Huber, Offenburg, Germany) with an accuracy of ± 0.1 °C. First, the background was taken using buffer (10 mM Hepes, pH 7.4) in the temperature range of 1–35 °C. Subsequently, to coat the silicon crystal, the cell was filled with 20 µl of the lipid stock solution, and the solvent was evaporated, resulting in a lipid multilayer film. For in situ measurements, the cell was subsequently filled with 20 µl buffer or ∆M3 peptide in buffer and incubated over the phase transition temperature for 15 min. For the evaluation of the gel–liquid-crystalline phase behavior, the peak position of the symmetric stretching vibration of the methylene band ν_s_(CH_2_) around 2970–2820 cm^−1^, which is a sensitive marker of lipid order, was taken. Furthermore, the vibrational band from the interface region associated with the ester carbonyl stretching around 1725 to 1740 cm^−1^ was analyzed.

To determine the position of the vibrational bands in the range of the second derivatives of the spectra, all the absorbance spectra in this range were cut and shifted to a zero baseline, and the peak picking function included in OPUS software was used. In each case, the results were plotted as a function of the temperature. To determine the transition temperature (T_m_) of the lipids, the curve was fitted according to the Boltzmann model to calculate the inflection point of the obtained thermal transition curves using the OriginPro 8.0 software (OriginLab Corporation, USA. The instrumental wavenumber resolution was better than 0.02 cm^*−*1^, and the wavenumber reproducibility in repeated scans was better than 0.1 cm^*−*1^.

## Results

### Antimicrobial and Bactericidal Activity of ∆M3

**Fig. 1:**
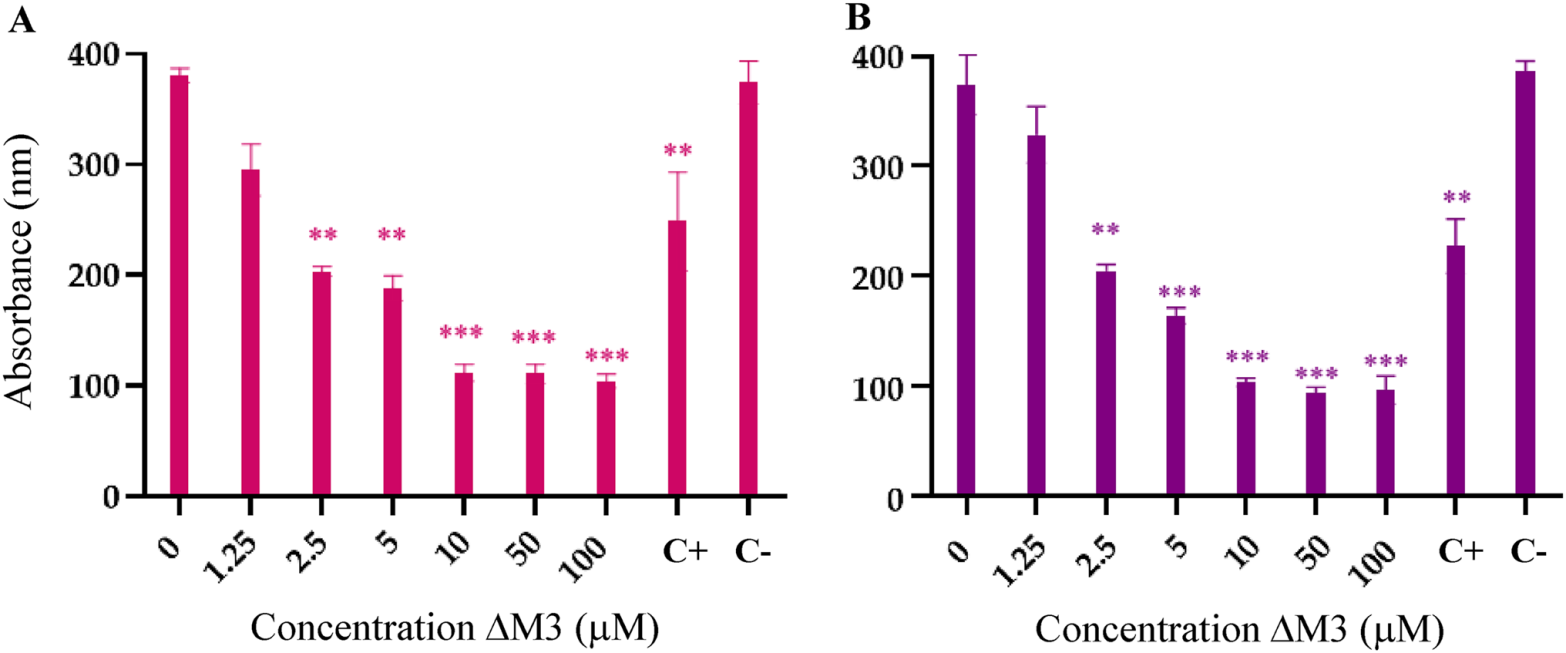
Antimicrobial activities of ΔM3 against **A** ESBL-producing *E. coli* isolates: HD7, HD8, HD11 BK43028, EB101, EB107, and EB127, and **B** *E. coli* ATCC 25922 strain. The differences with respect to non-treated isolates were obtained by one-way ANOVA where *p ≤ 0.05, **p ≤ 0.005, ***p ≤ 0.001

Using the broth microdilution assay, the antimicrobial activity of ΔM3 against seven ESBL-producing *E. coli* isolates and *E. coli* ATCC 25922 strain was performed. The obtained results are summarized in Fig. 1. The analysis of the results shows that ΔM3 has significant antimicrobial activity in all the tested isolates, including the ATCC strain. A detailed analysis of the data reveals that a MIC of 2.5 µM was obtained for the ΔM3 peptide, independently of the *E. coli* isolate or strain tested. It is important to highlight that the ΔM3 presented an antimicrobial activity similar to that obtained with the antibiotic Meropenem (positive control). In the absence of antibiotic or peptide treatment, no alteration in the bacterial viability of the cultures (negative control, PBS) was observed.

**Fig. 2:**
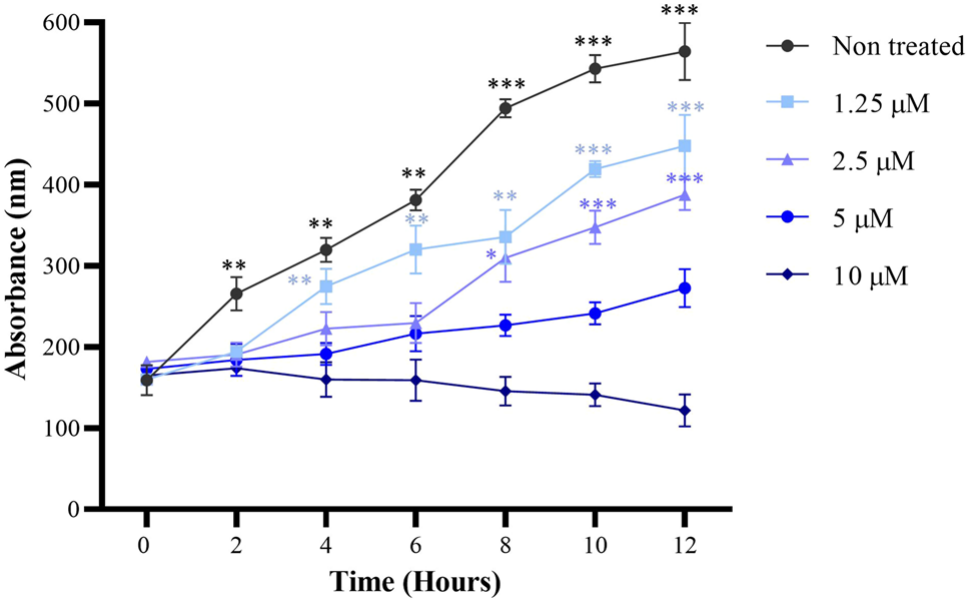
In vitro time-kill assessment of the bactericidal activity of ∆M3 on the exponential growth of ESBL-producing *E. coli* isolates within 12 h of exposure to different peptide concentrations. Each concentration was tested in triplicate for each hour and the data obtained are expressed as the mean ± standard deviation of three independent experiments. The differences with respect to non-treated isolates were obtained by one-way ANOVA where *p ≤ 0.05, **p ≤ 0.005, ***p ≤ 0.001

In order to determine the bactericidal effect of ∆M3 on ESBL-producing *E. coli* isolates, the peptide activity at different stages of microbial growth was evaluated. The results of the experiments are summarized in Fig. 2. The analysis of the results suggested that at a concentration lower than the MIC (1.25 µM) no significant effect on bacterial growth was observed. However, at the MIC (2.5 µM), a considerable decrease in the exponential growth of the isolates with respect to the untreated ones was found. Likewise, at 5 µM and 10 µM, a bactericidal effect was obtained on all the ESBL-producing *E. coli* isolates and on the control strain after 6 h of treatment. In this regard, after 12 h of incubation with the peptide, complete inhibition of bacterial growth was obtained at a concentration of 10 µM.

### Cytotoxic Effect of ∆M3 Against HaCaT Cells

**Fig. 3:**
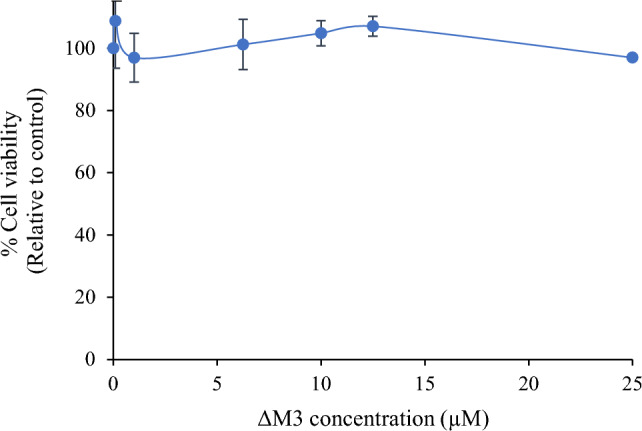
Cytotoxic effect of ΔM3 in HaCaT cells treated with different concentrations of peptide for 24 h. Values are expressed as the mean ± standard error of the mean (SEM) of three independent experiments. The differences with respect to non-treated cells were obtained by one-way ANOVA

The effect of ΔM3 on the viability of HaCaT human epidermic cells was studied as a measurement of the peptide toxicity towards higher-order eukaryotic skin cells. The results are summarized in Fig. 3. At all the tested concentrations, ∆M3 did not exhibit any cytotoxic effect on HaCaT cells. As a result, the peptide exhibited selectivity against bacteria while leaving HaCaT cells unaffected.

### Secondary Structure of ∆M3

The most accepted mechanism of action of AMPs is based on the interaction of the peptides with cell membranes. During the interaction, most peptides undergo a conformational change when they bind to cell membranes. This step has been considered as a crucial stage in the activity of AMPs. For this reason, it was proposed in this work to determine the secondary structure of ∆M3 in buffer and to evaluate the conformational change when the peptide was incubated with two representative lipid systems of erythrocytes and *E. coli* membranes. The results of the secondary structure analysis showed that ∆M3 presented a random coil structure in buffer and during the incubation with both multicomponent lipid systems (data not shown). The results highlight that ∆M3 did not suffer a conformational change when moving between the aqueous phase and the lipid environment.

### Membrane Permeability Experiments

**Fig. 4:**
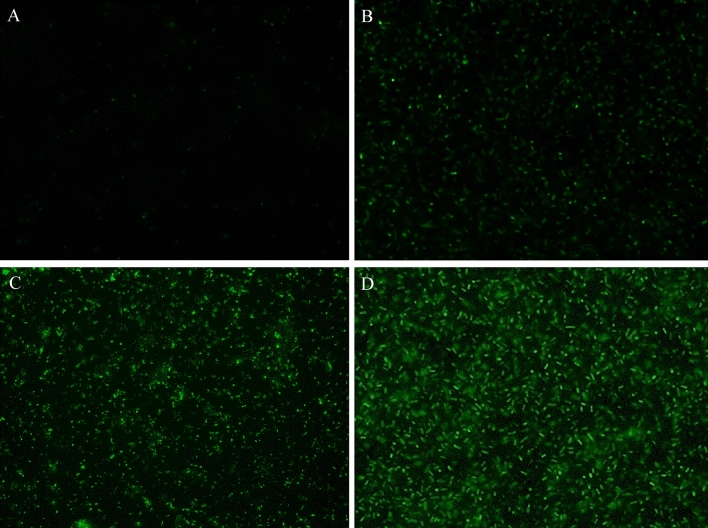
Cell permeability assay of ESBL-producing *E. coli* H8 isolate with the membrane-impermeant dye SYTOX® Green. Cells were treated with different concentrations of ΔM3: **A** Control, **B** 1.25 µM, **C** 2.5 µM, and **D** 5 µM

Figure 4 shows the results obtained when the ESBL-producing *E. coli* HD 8 isolate was incubated with different concentrations of ∆M3 peptide in the presence of the fluorophore SYTOX® Green. The analysis of the results indicates that there is a direct relationship between the increase in the peptide concentration and the increase in the fluorescence intensity. These results suggest a direct correlation between the increase in the concentration of ΔM3 and the loss of membrane integrity, evidencing the potential mechanism of the peptide related to the antimicrobial activity of ∆M3.

### Effect of ∆M3 on the Lipid Phase Transitions

**Fig. 5:**
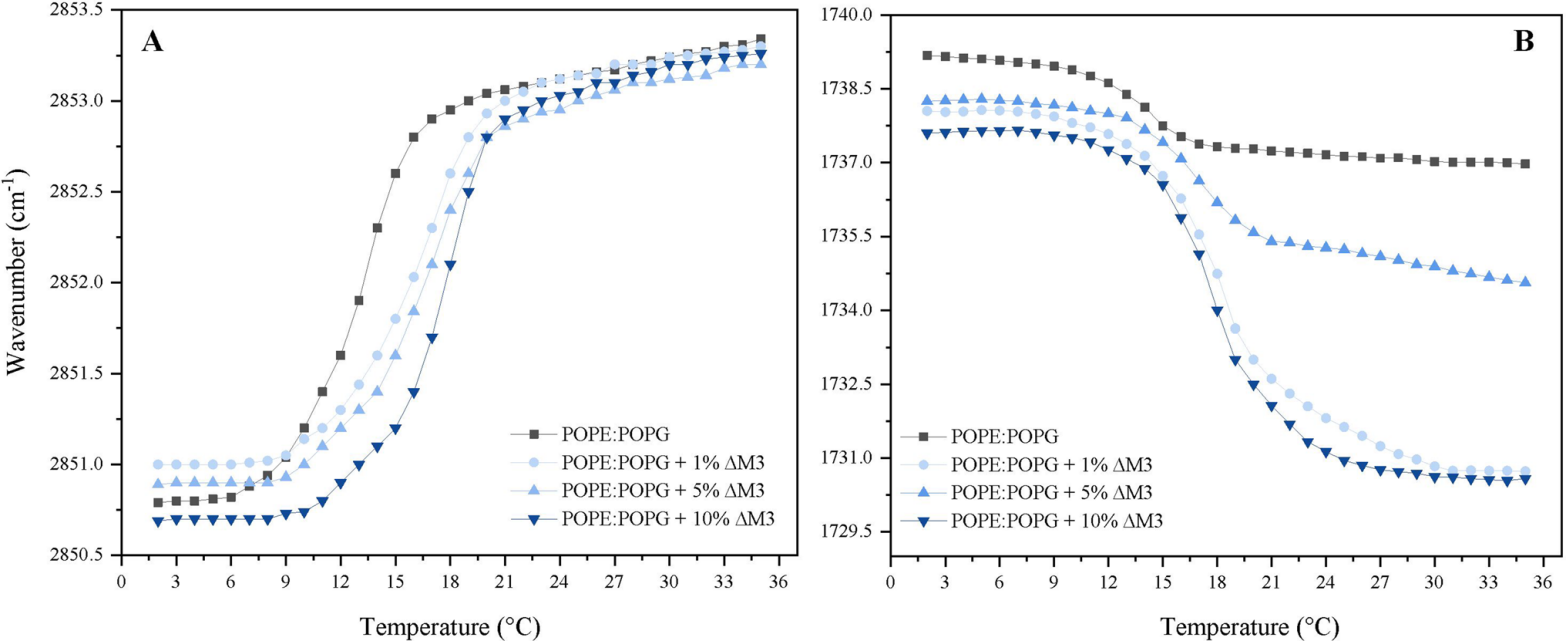
Peak positions of the **A** symmetric stretching vibration bands of the methylene groups and **B** stretching carbonyl vibration measured by FT-IR as a function of temperature. Effect of different concentrations of ΔM3 on the *E. coli* model membrane POPE:POPG (70:30)

One of the most sensitive techniques to monitor membrane dynamics is infrared spectroscopy. The changes induced by an exogenous molecule in the membrane can be monitored by following the different vibration bands of the functional groups of the lipids and their dependence on temperature. In this study, the effect induced by the ΔM3 on the phase transition temperature (T_m_) of SLBs built from POPE:POPG (70:30), representative of *E. coli* membranes, is summarized in Fig. 5A. The *E. coli* lipid system was built as the mixture POPE:POPG (70:30). For the pure lipids the T_m_ found were 25 °C and − 2 °C, respectively. However, when the lipids were in a 70:30 ratio, the T_m_ registered was 13.5 °C. This value corresponds to the second derivative of the curve obtained from the analysis of the phase transition in the absence of peptide. In the case of the lipid:peptide mixtures, analysis of the results shows that, as the concentration of the ΔM3 peptide increases, an increase in the transition temperature of the lipid systems was induced. At the lowest proportion of ΔM3, an increase of 3 °C in transition temperature was observed. Furthermore, at higher molar ratios of 10% of the peptide, a shift in the T_m_ was observed until it reached a T_m_ of 17 °C (Fig. 5A).

Additionally, with the goal of understanding whether ΔM3 induced changes in the lipid interface of the *E. coli* model membranes, the carbonyl group of the phospholipid was monitored as a sensitive sensor for the hydration of the lipids (Pérez et al. 2020; Watanabe et al. 2019). The C=O group of lipids is sensitive to hydration and shifts to lower wavenumbers when the water molecules interact via hydrogen bonds with the carbonyl group. Figure 5B shows the temperature dependence of the carbonyl stretching vibration of the *E. coli* model membrane. The analysis of the results demonstrated that ΔM3 strongly interacts with the lipid groups at the interface level; with increasing concentrations of the peptide the vibration of the C=O group is shifted to lower wavenumbers, demonstrating the increase of water molecules in the interface.

## Discussion

*Escherichia coli* is a bacterium that belongs to the *Enterobacteriaceae* family and is part of the natural intestinal microbiota of humans and animals. However, when it spreads to other areas of the body, it causes serious infections in the upper respiratory tract, urinary tract, surgical wounds, blood, and gastroenteritis (Larramendy et al. 2020; McDonald et al. 2021). The WHO in their report on antimicrobial resistance for the year 2022, emphasized the alarming growth of resistant *E. coli* isolates due to the presence of extended-spectrum *β*-lactamases (2022). The unfortunate association between resistance and the absence of effective antibiotics for treating infections makes the screening of new molecules with antimicrobial activity against ESBL-producing *E. coli* a priority.

The present study evaluated, through biological and biophysical tools, the potential activity of a synthetic peptide called ΔM3 against BL-producing *E. coli* and human epidermic cells. The results of the antimicrobial activity showed that the peptide is active against the seven tested ESBL-producing *E. coli* isolates in a dose-dependent manner. It is important to highlight that the MIC values obtained for ΔM3 did not vary significantly between the *E. coli* isolates evaluated, including the *E. coli* strain ATCC 25922 which is sensitive to antibiotics. Additionally, the MIC obtained with ΔM3 was much closer to that obtained with Meropenem, a recognized β-lactam antibiotic used in this study as a positive control. The results suggested that the activity of the ΔM3 peptide is not affected by the type of enzymatic resistance present in the *E. coli* isolates. ESBLs are β-lactamases capable of conferring bacterial resistance to penicillins, first-, second-, and third-generation cephalosporins, and aztreonam (not including cephamycins or carbapenems) by hydrolysis of these antibiotics. They are inhibited by antibiotics such as clavulanic acid. Therefore, the obtained results suggest that the mechanism by which ΔM3 exerts its biological activity could be associated with the interaction with bacterial membrane lipids. Our results of the antimicrobial activity of ΔM3 are in agreement with those obtained by Ebbensgaard, et al. in 2015. They compared a series of peptides from different origins against several microorganisms including resistant and sensitive *E. coli* isolates. The results showed that the peptides Cap18 Cap11 Cap11-1-18m2, Cecropin P1, Cecropin B, Indolicidin, Melittin, and Sub5 presented the same MIC value, independently of the resistant *E. coli* isolate evaluated (Ebbensgaard et al. 2015).

Regarding the role of the charge and its relationship with antimicrobial activity, numerous studies have shown that this parameter is fundamental to the mechanism of action of peptides (Fry 2018; Tan et al. 2021; Torres et al. 2019). ΔM3 has a charge of + 8, and a hydrophobicity of 45% at physiological conditions. The peptide sequence has positively charged Arginine and Lysine amino acids that facilitate the electrostatic attraction with the anionic groups of the bacterial membrane surface. These negatively charged groups belong to the lipids such as phosphatidylglycerol, conferring a negative surface charge to the membrane (Casares et al. 2019; Datta et al. 2016; Lee et al. 2019b; Pirtskhalava et al. 2021). On the other hand, while prokaryotic membranes are mostly composed of negatively charged phospholipids (Nowotarska et al. 2014; Travkova et al. 2017), the external monolayer of eukaryotic cell membranes are mainly composed of neutral phospholipids such as phosphatidylcholine and sphingomyelin. This difference between the bacterial and eukaryotic membranes could explain why ΔM3 showed no cytotoxicity on human epidermic cells, even at a concentration ten times higher than the MIC. In a previous work by our research group, the hemolytic activity of ΔM3 was evaluated. The results showed that ΔM3 has non-significant hemolytic activity (5%) at a concentration of 20 µM, which is eight times higher than the MIC obtained by *E. coli*. In terms of membrane composition, erythrocytes and HaCaT cells have a similar phospholipid composition, with the main lipids present being phosphatidylcholine, sphingomyelin, and phosphatidylethanolamine (Geng et al. 2023). Therefore, independently of the lipid proportions, both membranes are mostly zwitterionic or neutral, which considerably reduces the electrostatic initial interaction of the peptide with the membrane.

On the other hand, hydrophobicity plays an important role in the insertion of peptides into biological membranes (Chen et al. 2007; Yin et al. 2012), and is a factor that is directly related to the cytotoxicity of peptides (van der Weide et al. 2017). There are various studies that suggest that the relationship between the hydrophobicity and the charge of a peptide generates a balance between the insertion capacity of the peptide and the induction of lysis in the membranes, including the activity that the peptide may have in the host cells (Gong et al. 2019). For this reason, in this work, cytotoxic assays of ΔM3 in human epidermic cells were performed, in order to determine whether they have a harmful effect on human cells. Both cytotoxic and hemolytic assays have been used to determine the suitable concentration range of AMPs as potential new therapeutics to ensure their efficiency and safety (Peng et al. 2023). However, both assays demonstrated no interaction of ΔM3 with erythrocytes and epidermic cells.

It has been widely proposed that, after the initial step of electrostatic interaction, peptides undergo a conformational change from a random to a helical structure. However, the infrared spectroscopic experiments showed that the peptide did not suffer a conformational change during the interaction with the *E. coli* model membrane. It is important to highlight that the spectroscopic assays were performed in dynamic environments at a certain temperature, buffer, and in the presence of a representative lipid system of *E. coli* and erythrocyte cell membrane. Nevertheless, the FT-IR result is not unexpected, in previous research the ΔM3 structural studies were performed by circular dichroism using 2,2,2-trifluoroethanol (TFE) concluding that the peptide was not susceptible to adopt a helical structure (Guevara-Lora et al. 2023). Several peptides were demonstrated to be active without presenting a secondary structure. This is the case with indolicidin, a peptide with reported antibacterial activity against a wide range of microorganisms and Gram-positive and Gram-negative bacteria (Ando et al. 2010), with no secondary structure in solution or during interaction with membranes (Hsu & Yip 2007; Ladokhin et al. 1999). Another recognized peptide LTX-315, known as Oncopore™, a synthetic oncolytic peptide active in several cancer cell lines, has also been shown to be structureless in aqueous solution and model membranes made of 100% PC, 70/30% PC/PS, and 50% v/v TFE by circular dichroism (Koo et al. 2022). Our previous work with infrared spectroscopy also demonstrated the described outcome using tumoral and non-tumoral model membranes (Klaiss-Luna et al. 2023).

The cell membrane is responsible for the homeostasis of cells and microorganisms. It has a fundamental role in the transport of nutrients, waste elimination, and cellular communication (Yawata 2006). The loss of membrane integrity is an event that compromises the survival of the cell. For this reason, investigation of the effect of the synthetic peptide ΔM3 on the permeability changes of the *E. coli* membrane was essential in order to understand its potential relationship with the mechanism of action. The fluorescence microscopy results are based on the fact that the SYTOX® Green dye is internalized in the bacteria only when there is an alteration or damage to the membrane, subsequently binding to the DNA and emitting fluorescence (Shang et al. 2016; Teixeira et al. 2012). In the permeability tests carried out in this study, it was verified that ∆M3 caused the internalization of the fluorescent dye SYTOX® Green. The results suggest a mechanism based on an interaction with the *E. coli* membrane whereby antimicrobial activity is exerted.

Finally, with the aim of elucidating the interaction of ΔM3 with the *E. coli* membrane, FT-IR experiments were designed to monitor the vibrational changes of the methylene and carbonyl groups of the lipids as a function of temperature of a representative model of the *E. coli* membrane. The POPE:POPG model membrane has been extensively studied in order to understand the biophysical properties of the bacterial membranes. This binary systems is characterized for being a non-ideal mixture, due to the geometric and headgroup differences of both lipids, forming domains of the pure lipids (Navas et al. 2005). The results showed that the interaction of ΔM3 with the model membrane induced a change in the lipid phase transition of the SLBs. Therefore, the peptide induced an alteration of the methylene vibration from gel to crystalline liquid, a parameter associated with the order and packing of the hydrocarbon chains of lipids (Mantsch & McElhaney 1991). This interaction could be dominated by the electrostatic interaction of the positively charged ΔM3 with the negatively domains of the charged POPG, inducing a stronger de-mixing of the lipid species. Additionally, the carbonyl vibration also showed a higher hydration with increasing concentrations of the peptide. The results suggest that ΔM3 induces changes in the phase transition of the model synthetic membrane, causing an increase in the transition temperature, which correlates with the mechanisms of action that have been described for other PAMs with membrane-lytic activity (Park et al. 1998; Shai 1999). The increase in the phase transition temperature and in the interface, hydration may be associated with the electrostatic interaction of the peptide on the surface of the membrane and its subsequent insertion, restricting the characteristic mobility of lipids in the supported lipid bilayers and therefore increasing the rigidity of hydrocarbon chains and the reducing the carbonyl vibration. Numerous studies have used infrared spectroscopy techniques to investigate the effect of AMPs on membranes, with results showing that the interaction of AMPs with membrane lipids induces a structural change in the phospholipid bilayer that leads to cell death (Mantsch and McElhaney 1991). The results are directly related to those obtained previously by fluorescence microscopy, where a considerable alteration of the bacterial membrane in the *E. coli* isolates evaluated has been demonstrated through the emission of fluorescence, and with the results of the antimicrobial activity, where it was evidenced that the action of the peptides did not depend on the ESBL enzymes present in the *E. coli* isolates. The results demonstrate that the activity of the peptides is linked to their interaction with bacterial membrane lipids, which leads to destabilization of the bilayer and subsequent induction of cell death.

Polymyxin B is a peptide-type antibiotic used in clinical practice that is used as the last resort in treating infections caused by multi-resistant Gram-negative pathogens, despite notable side effects for patients such as neurotoxicity and nephrotoxicity (Simar et al. 2017). The mechanism of action of Polymyxin B, as well as that of many AMPs, is based on the alteration of the membrane and subsequent lysis of the microorganism (Lee and Lee 2015). The MIC reported by the CLSI for Polymyxin B is 2 µg/ml (equivalent to 1.5 µM). Taking these data into account, the peptide ∆M3 presents an MIC in the same range as that reported for Polymyxin B. However, although ∆M3 needs higher MICs to exert its antimicrobial activity, the cytotoxicity data suggests lower cytotoxicity.
